# Supplementary material for: Modeling somatic computation with non-neural bioelectric networks
Source: Sci Rep. 2019 Dec 9;9:18612. doi: 10.1038/s41598-019-54859-8 (PMC6901451; doi:10.1038/s41598-019-54859-8)
Supplement: Supplementary file 1 — Supplementary Information [file 41598_2019_54859_MOESM1_ESM.pdf]

“Modeling somatic computation with non-neural bioelectric networks”

Santosh Manicka and Michael Levin*

Allen Discovery Center
200 College Ave.
Tufts University
Medford, MA 02155

* Author for correspondence:
Email: michael.levin@tufts.edu
Tel.: (617) 627-6161

Supplementary information

1. Parameters of trained BEN models

All parameters used for the networks in this manuscript can be downloaded at https://gitlab.com/smanicka/BEN. The parameters of a BEN network that are learned during training, regardless of the problem they are trained to solve, are the 'weights' and 'bias' (highlighted in red in Fig. 8). The weights represent the strengths of the connections between cells (hence a matrix), determining the direction and speed by which the corresponding gap junctions alter their permeabilities dynamically as a function of V_{mem}. The biases represent the thresholds of the cells (hence a vector), determining how the signaling molecule switches speed and direction of change in concentration in the cells. The data files contain the weight and bias details of the best network from each of the five experiments described in the main text (simple AND logic gate, simple XOR logic gate, tissue-level AND, pattern detector and the compound NAND logic gate). File names are self-explanatory. Each file contains labels like 'Cell 1', 'Cell 2' etc. indicating the cell numbers. In the case of the compound NAND logic gate, the labels also indicate which module a cell belongs to (AND, BRIDGE or NOT), for example, 'AND Cell 1', 'BRIDGE Cell 10', 'NOT Cell 14' etc. The ordering convention adopted for numbering the cells is illustrated in Supplementary Fig. S1.

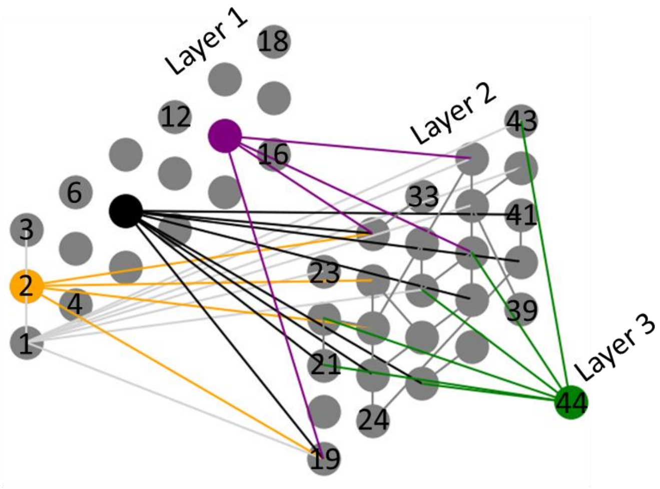

Supplementary figure S1. Numbering convention of the cells in a BEN network. Shown are a few examples of cell numbers in a sample network; the rest can be inferred. The lower left cell of the first layer is always numbered 1, while the highest numbered cell is the top right cell in the last layer.

2. BEN achieves robustness to damage by distributing information

Here, we present an analysis of the robustness of a nine-cell AND gate using the tools of information theory[125]. We wanted our network to be large enough to contain robustness characteristics (due to redundancy of function among the cells) but small enough to be amenable to analysis. First, we trained a nine-cell AND logic gate using backpropagation as described in the main text. Next, we knocked-off individual nodes that were neither input nor output cells, and then measured the performance of the resulting networks. Finally, we correlated the net amount of information about the inputs and outputs flowing through the rest of the nodes with the network performance upon removal of those nodes. We computed the information flow in the network using a Python package 'IDTxl'[125] that computes the conditional transfer entropy (the amount of

mutual information between a source's past state and a target's present state, given the target's past states) for every pair of nodes in the network (Supplementary Fig. S2).

We conclude that the network is more robust to removal of nodes through which less information flows (Supplementary Fig. S3). Moreover, there is modularity in the network, where some cells contain more information about one input versus the other (Supplementary Fig. S4). For example, cell 7 contains disproportionately more information about input 2 than input 1, while cells 2 through 6 contain slightly more information about input 1 compared to input 2. The biological implication is that real somatic tissues may also possess such robustness of function to damage of cells, and the mechanism by which this occurs may be distributed information processing, redundancy and modularity.

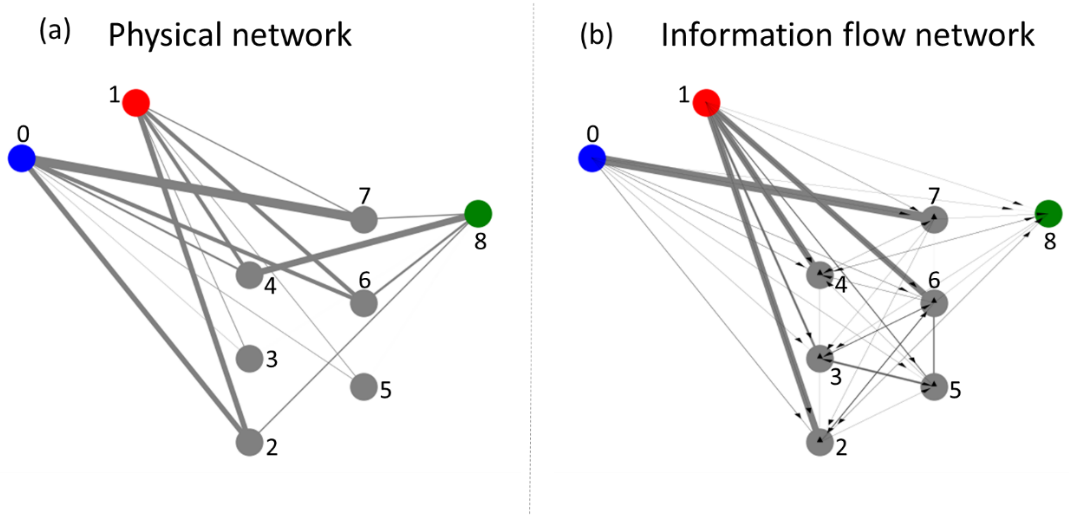

Supplementary figure S2. Physical and information-flow network depictions of a 9-cell AND gate. Color coding follows the same convention as in the main text. The edge weights of the physical network represent "meta weights" (see main text). The edges in the information-flow network are both weighted and directed: weights represent the amount of information-flow (measured by conditional transfer entropy) and directions represent the direction of the flow. Notice that corresponding weights are similar in the two networks, although there are some noticeable differences as well. Lastly, the information-flow network contains edges that are not present in the physical network; for every such edge an indirect path can be traced in the physical network.

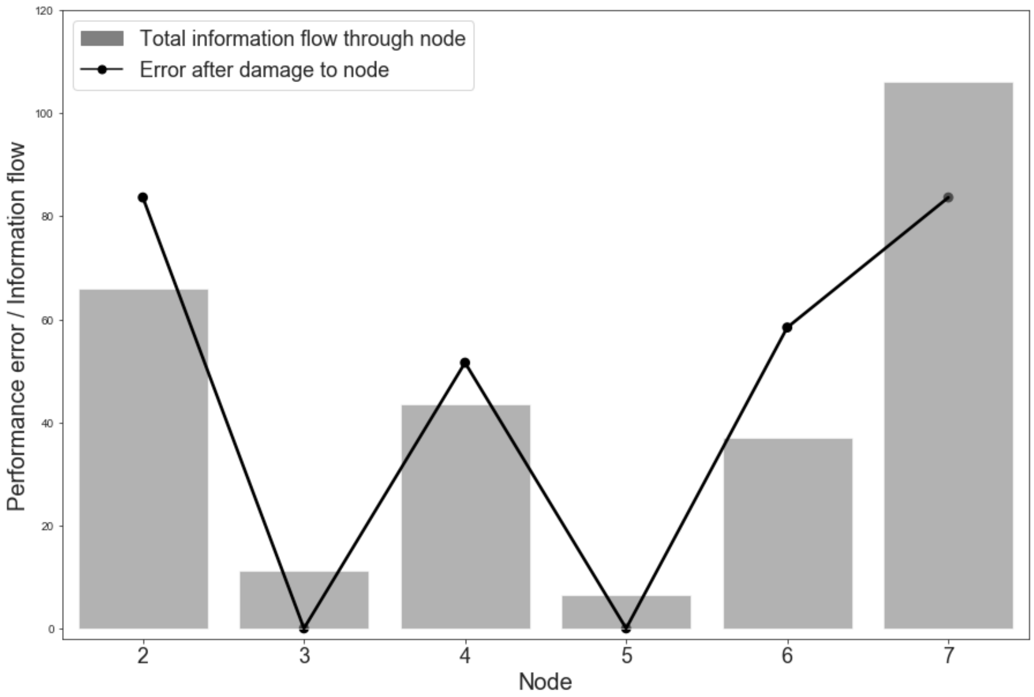

Supplementary figure S3. The relationship between information-flow and robustness to node damage. The heights of the bars represent the total amount of information about the inputs and output combined flowing through a node. Clearly, nodes that conduct more information are relatively more important to the functioning of the network: disabling them results in higher performance error of the whole network.

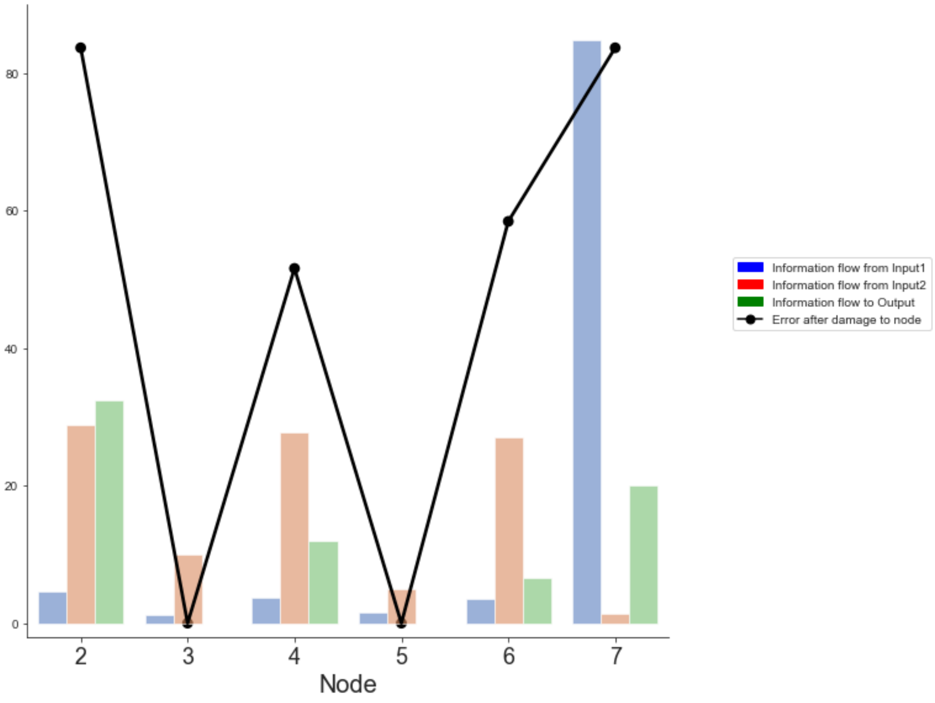

Supplementary figure S4. Modularity in information-flow. A form of specialization has emerged in this network: node 7 conducts disproportionately more information about input 1 compared to input 2 than any other intermediate cell, while all other cells conduct relatively more (but not disproportionately more) information about input 2 than about input 1. This segregation of knowledge about the inputs reflects the generic notion of modularity that biological systems are replete with both in terms of structure and function.

3. Alternative approaches to training BEN models

BPTT is just one of the many possible machine learning methods that can be used to train BEN models. To demonstrate that BEN networks can in principle be trained using other methods, we chose an alternative to gradient descent namely evolutionary optimization; genetic algorithms (GA) is an example of this class. Here, we used a GA to train a BEN network to function as an AND gate. Note that we didn't combine BP with GA for this example, unlike the tissue-level AND gates and the pattern detector discussed in the main text. We used the microbial GA (see 'Methods' section of the main text) with a population of 100 BEN networks, and a range of parameters including a recombination rate of 7-10%, a mutation rate of 5-10% and a deme size of 5-10. We found that none of the runs evolved a network to attain the optimum error (Supplementary Fig. S5), demonstrating the inferiority of GA compared to BPTT for training BEN networks. We then implemented a run by seeding the population with a previously best-known AND gate obtained through BPTT training, and injected Gaussian noise into the population. This led to the evolution of a BEN network that crossed the optimum error threshold (black line in Supplementary Fig. S5).

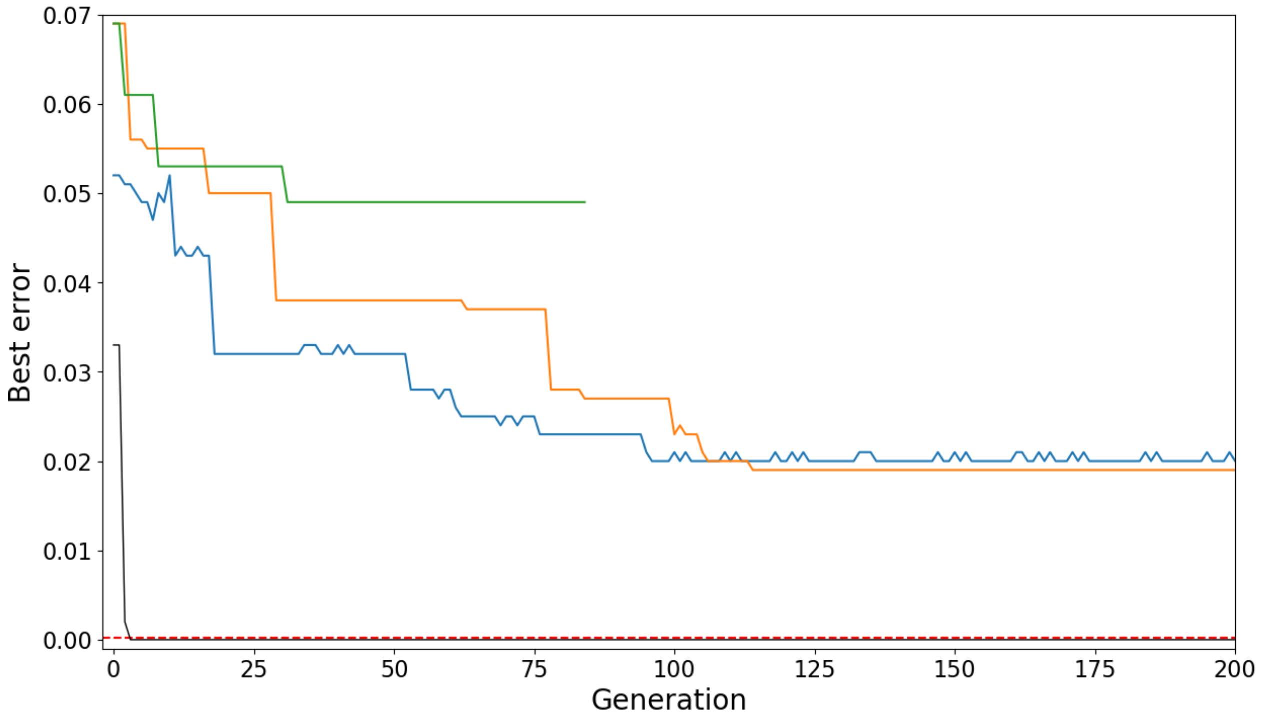

Supplementary figure S5. Performance errors of the best individuals in each iteration of the GA from four different runs indicated by the different colors. The black line represents a run with a population that was seeded with a previously trained (with BPTT) AND gate; Gaussian noise was injected into the population before initiating the run. The horizontal red dashed line indicates the optimum error required for a BEN network to qualify as a logic gate.

4. Designing and training a compound logic gate

Constructing a compound logic gate using pre-trained modules requires more than simply connecting the two modules. This is because, unlike genetic networks and neural networks which are directed, BEN networks are bidirectional due to the symmetric nature of gap junctions. Therefore, if the modules are simply connected together, then the output of one module will also receive signals from the input of the connected module. This could potentially result in the upstream module outputting the wrong state, and thus the downstream module receiving and

outputting the wrong states as well. To mitigate it, we included an “asymmetric bridge” layer (Supplementary Fig. S6) that interfaces the modules and trained it so that the compound gate as a whole behaves as desired: the downstream module outputs the correct state for every set of inputs received by the upstream module. The asymmetric aspect of the bridge lies in the differential connectivity to the upstream and downstream modules. First, only one node in the bridge is connected to the output of the upstream module, thus effectively creating a bottleneck between the two modules and minimizing the influence on the upstream output due to downstream noise. Second, all of the rest of the bridge nodes are connected to the input of the downstream modules=, thus maximizing the chances of passing down information reliably from the upstream module. For this particular experiment, we chose a bridge consisting of a single layer of 10 nodes connected as a chain. We explored various settings for training the NAND gate: (1) Specified the states of the inputs and outputs as in any other logic gate; (2) specified the states of the output and the input nodes of the upstream and downstream modules respectively; (3) trained the AND and NOT modules by accommodating intrinsic activity, as in the French-flag detector; and (4) trained the AND and NOT modules by outputting a V_{mem} of 0 mV when inputs are equal to 0 mV, thus creating a “scaffold” attractor for the modules to use as an intermediate step to overcome the effects of inter-module dynamics. We found that settings (3) and (4) were crucial to training the NAND gate. In particular, setting (4) manifests itself during the last phase of the simulation when both inputs are high; the output lingers around 0 mV (the scaffold attractor) for a while before “hammer-throwing” itself out of it into the correct attractor (time point 4 in Fig. 7).

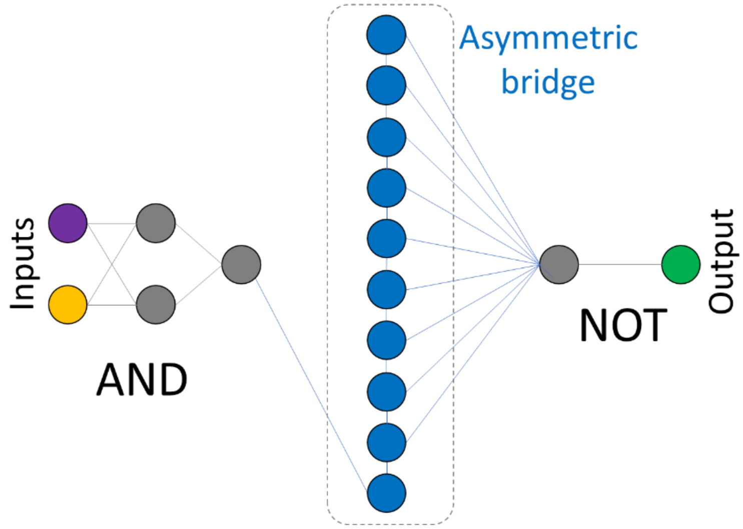

Supplementary figure S6. The architecture of a compound logic gate, specifically a NAND gate. The overall layout consists of three parts, namely the upstream (AND), the bridge and the downstream (NOT) modules. The AND and NOT modules are pre-trained, and only the bridge (blue) is trained here. The overall gate still consists of two inputs (orange and purple nodes) and a single output (green).
